# Electrodialysis Desalination with Simultaneous pH Adjustment Using Bilayer and Bipolar Membranes, Modeling and Experiment

**DOI:** 10.3390/membranes12111102

**Published:** 2022-11-04

**Authors:** Elena Nosova, Aslan Achoh, Victor Zabolotsky, Stanislav Melnikov

**Affiliations:** Faculty of Chemistry and High Technologies, Kuban State University, 350040 Krasnodar, Russia

**Keywords:** mathematical modeling, bipolar electrodialysis, pH adjustment, bipolar membrane, bilayer membrane, electrodialysis

## Abstract

A kinetic model of the bipolar electrodialysis process with a two-chamber unit cell formed by a bilayer (bipolar or asymmetric bipolar) and cation-exchange membrane is proposed. The model allows describing various processes: pH adjustment of strong electrolyte solutions, the conversion of a salt of a weak acid, pH adjustment of a mixture of strong and weak electrolytes. The model considers the non-ideal selectivity of the bilayer membrane, as well as the competitive transfer of cations (hydrogen and sodium ions) through the cation-exchange membrane. Analytical expressions are obtained that describe the kinetic dependences of pH and concentration of ionic components in the desalination (acidification) compartment for various cases. Comparison of experimental data with calculations results show a good qualitative and, in some cases, quantitative agreement between experimental and calculated data. The model can be used to predict the performance of small bipolar membrane electrodialysis modules designed for pH adjustment processes.

## 1. Introduction

One of the important characteristics of water quality is pH [1]. Any industry that uses water must control the pH level to meet technological and sanitary standards. Usually, acid or base dosing is used to adjust the pH, which leads to the appearance of ballast ions in solutions, which must subsequently be removed. This method is widely used to reduce the acidity of fruit juices [2,3], to isolate proteins [4], and in fine chemical synthesis [5,6]. As an alternative to direct acid dosing, ion-exchange can be used. Ion-exchange is used for tartrate stabilization of wine [7,8,9,10], control of fruit juice acidity [11,12], an in casein production [13]. The disadvantage of using ion-exchange resins is the need for their periodic regeneration.

Methods using electromembranes for pretreatment and processing of solutions, for example, electrodialysis (ED) can run continuously without the need for periodical regeneration. During electrodialysis, the ionic components are removed by the action of an electric field. As a result, two streams are formed: a solution purified from ionic impurities and a concentrate into which they have passed. Conventional electrodialysis can be used to control the pH of the medium in the food industry (fruit juices, wine, whey, etc.) [14,15,16,17].

In addition to conventional electrodialysis, electrodialysis with bipolar membranes (BMED) is widely used [18,19,20]. Bipolar membranes are a special class of ion exchange membranes, the main function of which is the generation of H^+^/OH^−^ ions from water molecules under the action of an electric field. Bipolar membranes consist of a cation-exchange layer and an anion-exchange layer joined together is series [21]. Some researchers also specifically mention the intermediate layer, which can perform several functions: connect monopolar layers and/or contain a catalyst for the water dissociation reaction [22]. Numerous studies and reviews have been devoted to the use of bipolar electrodialysis in general [21,23] and in the food industry [24,25,26,27]. The processes of isolating casein [28], adjusting the pH of whey [29], separating proteins, neutralizing the acidity of fruit juices [30,31] have been studied. Bipolar electrodialysis is used to obtain organic acids [32,33,34], with a significant change in pH. When processing weak acids, a two-chamber cell configuration with bipolar and cation-exchange membranes is used [35]. It is not clear when cation-exchange membrane properties play a crucial role in the overall process characteristics. Novel composite cation-exchange membranes [36] can be suitable for such applications.

When adjusting the pH of dilute solutions, it is possible to use asymmetric bipolar membranes. The main difference between asymmetric bipolar membranes and classical bipolar membranes, which have the same thickness of monopolar layers, is a much more significant nonselective transfer of salt ions, due to which such membranes allow simultaneous desalting of the solution along with pH adjustment [37].

The possibility of combining two functions (removal of salt ions and pH adjustment) using a single-layer mosaic membrane (contains cation and anion exchange sites) was described in a patent by Kollsman [38]. For the first time, desalination of a sodium chloride solution with simultaneous adjustment of the pH of the medium by electrodialysis using asymmetric bipolar membranes was proposed by Leitz [39].

Grebenyuk et al. [40] obtained “semi-bipolar” membranes by electrodeposition of an anion-exchange resin suspension onto a cation-exchange membrane-substrate. In [41], the authors showed a high charge selectivity of the obtained membranes at super-limiting current densities, and in [42], to reduce the flux of salt ions, the authors reduced the porosity of the layer of electrodeposited ion exchangers by introducing polyelectrolytes into the coating. The disadvantage of these asymmetric bipolar membranes was their low mechanical stability.

Russell and Samuel [43] were the first to use the phase inversion method to obtain asymmetric bipolar membranes. This approach allowed varying the thickness of the modifying layer.

Stratmann et al. [44], Balster et al. [45], and Xu et al. [46,47,48] studied the effect of the thickness and exchange capacity of a thin layer on the electrochemical characteristics of asymmetric bipolar membranes.

The authors of [49] showed that the functional properties of asymmetric bipolar membranes are determined by the thickness of the modifying film and the concentration of the solution. At a small film thickness, the diffusion of salt ions is dominant in the processes occurring in asymmetric bipolar membranes in moderately concentrated solutions. When the film thickness increases or the concentration of the electrolyte solution decreases, the water dissociation reaction becomes the dominant process. In this case, the asymmetric bipolar membrane can be no worse than traditional bipolar membranes. In intermediate situations, the process of diffusion transfer of salt ions and the chemical reaction of water dissociation can be comparable with each other. The latter conditions make it possible to carry out the process of “reaction electrodialysis” for the separation of strong and weak electrolytes [50].

Currently, research is aimed at improving the efficiency of bipolar and asymmetric bipolar membranes and electrodialysis processes using them. Mathematical modeling can be used to describe the electrodialysis process (including those with bilayer membranes). The developed mathematical models can consider several complex phenomena arising in electromembrane systems: ion-exchange equilibrium, concentration polarization, diffusion, and mass transfer phenomena, etc.

Most of the existing mathematical models are aimed at describing mass transfer during the desalination of solutions by electrodialysis and calculating its productivity [51,52,53,54,55,56]. Some of the works consider specific processes of electrodialysis: purification of solutions containing heavy metal ions [57,58], synthesis of sodium sulfate by metathesis electrodialysis [59], electrodialysis concentration [60], separation of strong and weak acids [61,62] or phenomena, which affect the transfer of ions through ion-exchange membranes, such as water dissociation and electroconvection [63,64,65].

Mathematical modeling of the bipolar electrodialysis process is the subject of various works that describe the dissociation of water at the bipolar boundary [66,67], the evaluation of the efficiency of the electrodialysis modules [68], the transport of ions through a bipolar membrane [69], and factors affecting the kinetics of the process [70,71,72]. Most studies deal with the conversion of strong electrolytes (sodium chloride, sodium nitrate, sodium sulfate) into the corresponding acids and bases [73] or special cases of obtaining weak organic acids [74,75,76].

An experimental study and numerical simulation of the pH adjustment process was carried out by Zabolotskii et al. for a system containing only a strong electrolyte [77] and for a system containing weak electrolyte ions (hydrocarbonate ions) [78].

This paper presents the results of tests of laboratory electrodialyzers designed for desalination of solutions with simultaneous pH adjustment using bilayer (bipolar and asymmetric bipolar) membranes. For a theoretical description of the process, analytical expressions will be obtained that describe the kinetic curves of changes in the pH and the concentration of salt ions.

## 2. Materials and Methods

### 2.1. Laboratory Electrodialysis Setup

**Figure 1:**
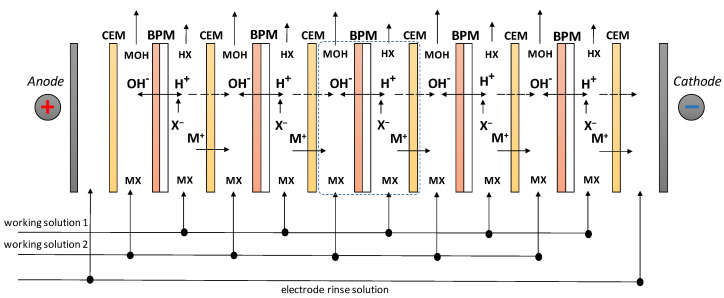
Schematic depiction of the membrane stack.

The study of the pH adjustment process was carried out in a laboratory electrodialyzer (Figure 1). The membrane stack of the electrodialyzer consisted of five elementary cells. Two types of elementary cells were studied: a two-chamber cell “bipolar membrane/cation exchange membrane” (for cases I, III, IV) and “bilayer membrane/cation exchange membrane” (for cases II, V). Ralex CM-Pes membranes (Mega a.s., Strazh pod Ralskem, Chezch Republic) were used as cation-exchange membranes in all experiments. A MB-2m laboratory bipolar membrane [79] was used as a bipolar membrane. Bilayer membranes were BM-ac-2K and BM-a-30 membranes [37]. Bipolar and bilayer membranes were oriented with the cation-exchange layer towards the cathode, and the anion-exchange layer towards the anode, to observe the reverse bias conditions.

Polyethylene frames were placed between the membranes, separating the membranes from each other, and setting the direction of the solution flow through the channel. The thickness of each frame was 0.9 mm. The working area of each frame was 60 × 15 mm^2^, and the external dimensions were 90 × 25 mm^2^. Working solutions were fed in parallel to the compartments for desalination (acidification) and concentration (alkalization). To intensify the mixing of solutions and reduce the effect of diffusion boundary layers on the transport properties of membranes, polyethylene mesh separators (porosity 0.85) were located between the membranes inside frames.

**Figure 2:**
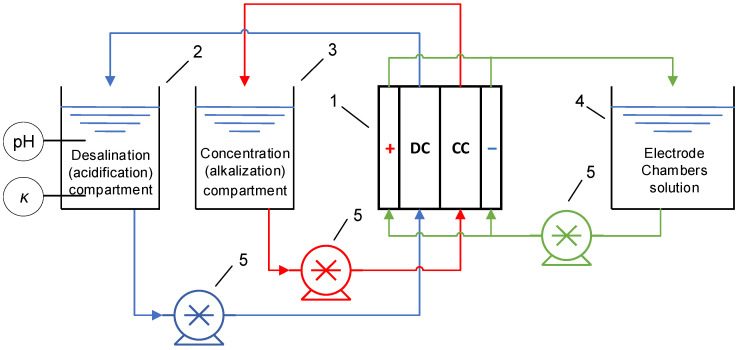
Hydraulic scheme of the electrodialysis setup. 1—electrodialysis module, 2—desalination (acidification) compartment tank, 3—concentration (alkalization) compartment tank, 4—electrode rinse solution tank, 5—pumps.

The hydraulic scheme of the experimental setup is shown on the Figure 2.

The volumetric flow rate of the solutions supplied to the working chambers were 14 L/h (linear velocity 6.8 cm/s) and 12 L/h (linear velocity 6.5 cm/s) for the electrode chambers. A sodium sulfate solution with a concentration of 0.02 mol-eq/L was used as the electrode rinse solution in all experiments.

#### 2.1.1. Reagents and Analytics

Sodium chloride, sodium acetate, sodium hydroxide and hydrochloric acid were purchased from JSC “Vecton” (JSC “Vecton”, Krasnodar, Russia). All reagents were of analytical grade. All reagents were used as is without purification.

In all experiments, the pH and conductivity of solutions were measured using Expert-001 pH and conductivity meters (JSC “Ekonics-expert”, Moscow, Russia).

In Section 4.1, the concentrations of sodium and chloride ions were measured using ion-chromatography on Stayer chromatographer (JSC “Akvilon”, Moscow, Russia).

In Section 4.2, the concentration of acidic acid was measured by direct potentiometric titration.

In Section 4.3, the concentrations of chloride and acetate ions were measured using titration technique described previously [50]. The sodium ion concentration was further calculated from electroneutrality condition.

#### 2.1.2. Integral Current Efficiency

Integral (mean over time) current efficiency was calculated as:

(1) $$\eta_{i}^{\mathrm{int}}=\pm \frac{FV}{iSn}\frac{\left(c_{i}(0)-c_{i}(\tau )\right)}{\tau }.$$

where *F* is the Faraday constant, A·s/mol; *V* is the volume of the solution in the compartment, L; $c_{i}(0),\;c_{i}(\tau )$ are the concentrations of component *i* at the beginning of the experiment and at time τ, mol/L; *i* is the current density, A/dm^2^; *S* is the membrane area, dm^2^; *n* is the number of elementary cells in the membrane stack; τ is the time from the beginning of the experiment at which the measurement was carried out.

### 2.2. Membranes

Ralex CM-Pes is a heterogeneous cation exchange membrane based on sulfonated polystyrene cross-linked with divinylbenzene. The membrane is produced by hot rolling of a mixture consisting of a finely dispersed ion-exchange powder and polyethylene in an approximate ratio of 2:1. The physicochemical properties of the Ralex CM-Pes membranes are presented in the Table 1.

The MB-2m is a tailor-made heterogeneous bipolar membrane [79]. The membrane is obtained by hot pressing of heterogeneous industrial Ralex CMH cation-exchange membrane and Ralex AMH anion-exchange membrane. The catalytic additive of ion-polymer containing phosphoric acid ionic groups is applied to the surface of the cation-exchange membrane before pressing. The catalytic powder is applied to the surface of the cation exchange membrane as a wet paste to a thickness of 0.2 mm. The mass the powder applied is 0.8 mg/cm^2^.

BM-a-30 bilayer membranes were obtained by applying a thin layer of sulfonated polytetrafluoroethylene to the surface of the Ralex AMH-Pes membrane at a rate of 0.025 cm^3^/cm^2^. As a result, membranes with a cation-exchange layer thickness of 25 ± 2 μm were obtained. A solution of sulfonated polytetrafluoroethylene was prepared by mixing concentrated anhydrous acetic acid and a 10 vol.% solution of sulfonated polytetrafluoroethylene in isopropyl alcohol in a ratio of 1:1. To improve the adhesion between the cationic and anion exchange layers, the surface of the substrate membrane was preliminarily treated with a solution of acetic acid.

BM-ac-2K bilayer membranes were obtained by applying a modifier solution (a solution of the same composition as in the preparation of BM-a-30 membranes) containing a micro-dispersion of a catalyst for the reaction of water dissociation—a phosphoric acid cation exchanger—on the surface of the Ralex AMH-Pes membrane in an amount of 2 mg/cm^2^.

The physicochemical properties of the bilayer membranes are presented in the Table 2.

Before testing, the membranes underwent standard pretreatment [80]. All membranes were stored in the working solution.

## 3. Kinetic Model of the Electrodialysis Process

**Figure 3:**
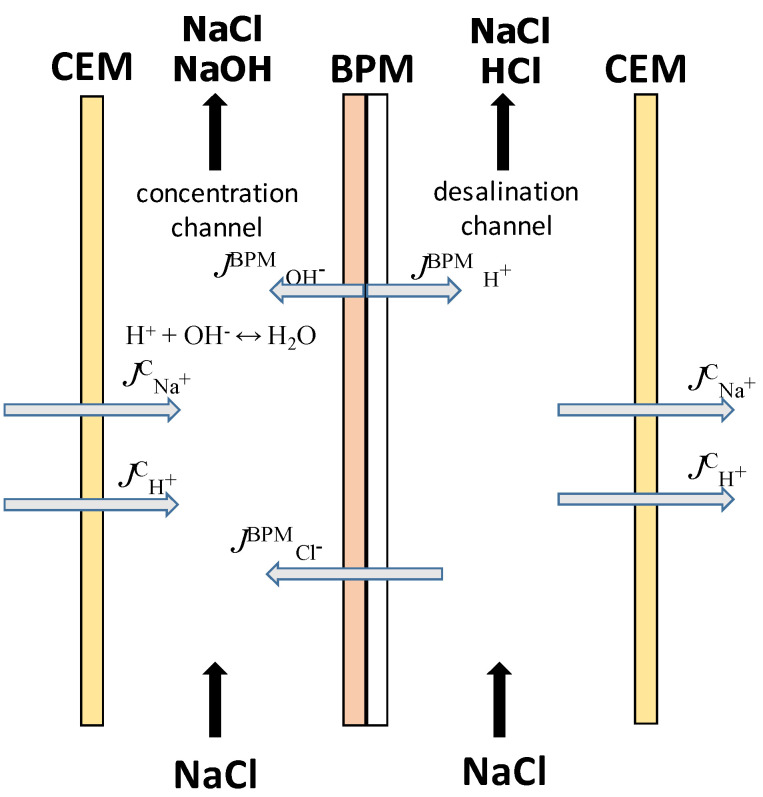
Scheme of ions fluxes in the studied electromembrane system.

The investigated electromembrane system consists of two channels—a desalting channel and a concentration channel, limited by a bilayer (or bipolar) and cation-exchange membranes (Figure 3).

The fluxes of ions in an electromembrane system is given by the Nernst–Planck equation (written for the one-dimensional case):

(2) $$J_{i}^{}=-D_{i}\left(\frac{dc_{i}}{dx}+\frac{z_{i}F}{RT}c_{i}\frac{d\varphi }{dx}\right).$$

The flux of hydrogen ions in the desalination chamber is defined as the difference between the fluxes of hydrogen ions generated by the bilayer membrane ($J_{H}^{\mathrm{BM}}$) and hydrogen ions passing through the cation exchange membrane into the concentration chamber ($J_{H}^{C}$):

(3) $$J_{H}^{\mathrm{dc}}=V^{\mathrm{dc}}(\tau )\frac{dc_{H}^{\mathrm{dc}}}{d\tau }=J_{H}^{\mathrm{BM}}-J_{H}^{C}.$$

The flux of hydroxide ions in the alkaline chamber is also related to the flux of hydrogen ions through the cation exchange membrane (due to water recombination reaction):

(4) $$J_{\mathrm{OH}}^{\mathrm{cc}}=V^{cc}(\tau )\frac{dc_{\mathrm{OH}}^{\mathrm{cc}}}{d\tau }=J_{\mathrm{OH}}^{\mathrm{BM}}-J_{H}^{C}.$$

During electrodialysis, the concentration of salt ions (Na^+^ and Cl^−^) in the desalination chamber decreases, while in the concentration chamber it increases. For the fluxes of sodium ions and chloride ions, the following expressions can be written:

(5) $$V^{\mathrm{dc}}(\tau )\frac{dc_{\mathrm{Na}}^{\mathrm{dc}}}{d\tau }=-J_{\mathrm{Na}}^{C},$$

(6) $$V^{\mathrm{cc}}(\tau )\frac{dc_{\mathrm{Na}}^{\mathrm{cc}}}{d\tau }=J_{\mathrm{Na}}^{C},$$

(7) $$V^{\mathrm{dc}}(\tau )\frac{dc_{\mathrm{Cl}}^{\mathrm{dc}}}{d\tau }=-J_{\mathrm{Cl}}^{\mathrm{BM}},$$

(8) $$V^{\mathrm{cc}}(\tau )\frac{dc_{\mathrm{Cl}}^{\mathrm{cc}}}{d\tau }=J_{\mathrm{Cl}}^{\mathrm{BM}}.$$

The recombination reaction of hydrogen and hydroxyl ions is fast, and considering that $J_{\mathrm{OH}}^{\mathrm{BM}}>J_{H}^{C}$, the change in the concentration of hydrogen ions in the concentration chamber and hydroxide ions in the desalting chamber can be neglected:

(9) $$V^{\mathrm{dc}}(\tau )\frac{dc_{\mathrm{OH}}^{\mathrm{dc}}}{d\tau }=0.$$

(10) $$V^{cc}(\tau )\frac{dc_{H}^{cc}}{d\tau }=0,$$

We also accept the following relationships between fluxes:

(11) $$J_{H}^{\mathrm{BM}}=J_{\mathrm{OH}}^{\mathrm{BM}},$$

(12) $$J_{H}^{C}+J_{\mathrm{Na}}^{C}=J_{H}^{\mathrm{BM}},$$

(13) $$J_{\mathrm{OH}}^{\mathrm{BM}}+J_{\mathrm{Cl}}^{\mathrm{BM}}=\frac{i}{F}.$$

The following relation interconnects the fluxes of hydrogen and sodium ions through the cation-exchange membrane:

(14) $$\frac{J_{\mathrm{Na}}^{C}}{J_{H}^{C}}=P_{Na/H}(i)\frac{c_{\mathrm{Na}}^{\mathrm{dc}}}{c_{H}^{\mathrm{dc}}},$$

where $P_{Na/H}(i)$ is the specific permeability coefficient of the cation-exchange membrane in the sodium/proton pair at a given current density. This coefficient depends on the current flowing in the system, and this dependence is non-linear [81].

The conditions of material balance and electroneutrality impose the following restrictions on the concentration of ions in the channels:

(15) $$V^{\mathrm{dc}}(\tau )c_{\mathrm{Na}}^{\mathrm{dc}}(\tau )+V^{\mathrm{cc}}(\tau )c_{\mathrm{Na}}^{\mathrm{cc}}(\tau )=V^{\mathrm{dc}}(0)c_{\mathrm{Na}}^{\mathrm{dc}}(0)+V^{\mathrm{cc}}(0)c_{\mathrm{Na}}^{\mathrm{cc}}(0),$$

(16) $$V^{\mathrm{dc}}(\tau )c_{\mathrm{Cl}}^{\mathrm{dc}}(\tau )+V^{\mathrm{cc}}(\tau )c_{\mathrm{Cl}}^{\mathrm{cc}}(\tau )=V^{\mathrm{dc}}(0)c_{\mathrm{Cl}}^{\mathrm{dc}}(0)+V^{\mathrm{cc}}(0)c_{\mathrm{Cl}}^{\mathrm{cc}}(0),$$

(17) $$c_{\mathrm{Na}}^{\mathrm{dc}}(\tau )+c_{H}^{\mathrm{dc}}(\tau )=c_{\mathrm{Cl}}^{\mathrm{dc}}(\tau )+c_{\mathrm{OH}}^{\mathrm{dc}}(\tau ),$$

(18) $$c_{\mathrm{Na}}^{\mathrm{cc}}(\tau )+c_{H}^{\mathrm{cc}}(\tau )=c_{\mathrm{Cl}}^{\mathrm{cc}}(\tau )+c_{\mathrm{OH}}^{\mathrm{cc}}(\tau ).$$

In both channels, the condition of chemical equilibrium is also satisfied at any point in time:

(19) $$c_{H}^{\mathrm{dc}}(\tau )c_{\mathrm{OH}}^{\mathrm{dc}}(\tau )=K_{w},$$

(20) $$c_{H}^{\mathrm{cc}}(\tau )c_{\mathrm{OH}}^{\mathrm{cc}}(\tau )=K_{w}.$$

In the general case, the volumes of solutions in each of the channels are variable, which is associated both with the reaction of water dissociation on the bilayer membrane and with the transfer of water as part of the hydration shells of ions. As a result, the volume of the solution in the desalination channel ($V^{\mathrm{dc}}(\tau )$) decreases, while the volume of the solution in the concentration chamber ($V^{\mathrm{cc}}(\tau )$) increases. The change in the volume of solutions can also be expressed in terms of ion fluxes, considering the hydration number of salt ions and the osmotic permeability of membranes; however, it is easier to do this by introducing some constant *r*, which considers the total contribution of all water transport mechanisms in the channels:

(21) $$V^{\mathrm{dc}}(\tau )=V^{\mathrm{dc}}(0)-r\tau ,$$

(22) $$V^{\mathrm{cc}}(\tau )=V^{\mathrm{cc}}(0)+r\tau .$$

If the initial volumes in each of the compartments are equal, then:

(23) $$V^{\mathrm{dc}}(\tau )+V^{\mathrm{cc}}(\tau )=2V(0)$$

The solution of the system of Equations (2)–(23) in general form can only be obtained numerically, since the ion fluxes (Equations (2)–(10)) are described by nonlinear differential equations.

Below we will analyze several practical cases when, by introducing reasonable assumptions, it is possible to obtain an analytical solution of the indicated system of equations. For simplicity, we will only consider the desalination (acidification) channel.

### 3.1. Ideally Selective Bipolar Membrane in Strong 1-1 Electrolyte Solution (Case I)

Consider a system operating in a circulating mode. Sodium chloride solution circulates in the desalination and concentration paths. The current flowing in the system exceeds the limiting electro-diffusion current on the cation exchange membrane. Let us neglect the change in the volume of the solution in the paths of the electrodialyzer over time. We will consider only the processes occurring in the desalination chamber. Let the initial pH of the solution in the desalination chamber be known. The initial concentration of the electrolyte is also known.

In subsequent cases, only conditions that differ from this base case will be specified.

The overlimiting current mode makes it possible to transform Equation (14) into the following form [81]:

(24) $$\frac{J_{\mathrm{Na}}^{C}}{J_{H}^{C}}=\frac{D_{\mathrm{Na}}}{D_{H}}\frac{c_{\mathrm{Na}}^{\mathrm{dc}}(\tau )}{c_{H}^{\mathrm{dc}}(\tau )}.$$

An ideally selective bipolar membrane does not allow any ions to pass through, except for the products of water dissociation, then Equations (11)–(13) can be converted to the following form:

(25) $$J_{\mathrm{Cl}}^{\mathrm{BM}}=0,$$

(26) $$J_{H}^{\mathrm{BM}}=J_{\mathrm{OH}}^{\mathrm{BM}}=\frac{i}{F},$$

(27) $$J_{H}^{C}+J_{\mathrm{Na}}^{C}=\frac{i}{F}.$$

By jointly solving (24) and (27) with the subsequent substitution of the result into Equation (5), we obtain (taking into account the constant volume):

(28) $$V^{\mathrm{dc}}\frac{dc_{\mathrm{Na}}^{\mathrm{dc}}}{d\tau }=-\frac{i}{F}\frac{D_{\mathrm{Na}}c_{\mathrm{Na}}^{\mathrm{dc}}}{D_{\mathrm{Na}}c_{\mathrm{Na}}^{\mathrm{dc}}+D_{H}c_{H}^{\mathrm{dc}}}.$$

Considering the ideal selectivity of the bipolar membrane, it can be imagined that sodium ions are gradually replaced by hydrogen ions in the desalination chamber, while the number of positively charged ions remains constant. If chemical reactions with the participation of hydrogen ions also do not occur in the system, then from the condition of electrical neutrality and material balance it follows:

(29) $$c_{\mathrm{Na}}^{\mathrm{dc}}(\tau )+c_{H}^{\mathrm{dc}}(\tau )=c_{\mathrm{Na}}^{\mathrm{dc}}(0)+c_{H}^{\mathrm{dc}}(0).$$

Expressing $c_{H}^{\mathrm{dc}}(\tau )$ from Equation (29) and substituting into Equation (28) after permutation, separation of variables and integrating, taking into account that for τ=0, $c_{\mathrm{Na}}^{\mathrm{dc}}=c_{\mathrm{Na}}^{\mathrm{dc}}(0)$ and for some τ>0, $c_{\mathrm{Na}}^{\mathrm{dc}}=c_{\mathrm{Na}}^{\mathrm{dc}}(\tau )$ we obtain:

(30) $$\left(D_{\mathrm{Na}}-D_{H}\right)c_{\mathrm{Na}}^{\mathrm{dc}}(\tau )+D_{H}\left(c_{\mathrm{Na}}^{\mathrm{dc}}(0)+c_{H}^{\mathrm{dc}}(0)\right)\ln\left(c_{\mathrm{Na}}^{\mathrm{dc}}(\tau )\right)=D^{I},$$

where

(31) $$D^{I}=\left(D_{\mathrm{Na}}-D_{H}\right)c_{\mathrm{Na}}^{\mathrm{dc}}(0)+D_{H}\left(c_{\mathrm{Na}}^{\mathrm{dc}}(0)+c_{H}^{\mathrm{dc}}(0)\right)\ln\left(c_{\mathrm{Na}}^{\mathrm{dc}}(0)\right)-\frac{iD_{\mathrm{Na}}}{V^{\mathrm{dc}}F}\tau .$$

We obtain an analytical expression for $c_{\mathrm{Na}}^{\mathrm{dc}}(\tau )$ using the Lambert W-function [82]:

(32) $$c_{\mathrm{Na}}^{\mathrm{dc}}(\tau )=\frac{D_{H}\left(c_{\mathrm{Na}}^{\mathrm{dc}}(0)+c_{H}^{\mathrm{dc}}(0)\right)}{D_{\mathrm{Na}}-D_{H}}W\left(z^{I}\right),$$

where $W\left(z^{I}\right)$ is the Lambert *W*-function of argument *z^I^*:

(33) $$z^{I}=\frac{D_{\mathrm{Na}}-D_{H}}{D_{H}\left(c_{\mathrm{Na}}^{\mathrm{dc}}(0)+c_{H}^{\mathrm{dc}}(0)\right)}\exp\left[\frac{D^{I}}{D_{H}\left(c_{\mathrm{Na}}^{\mathrm{dc}}(0)+c_{H}^{\mathrm{dc}}(0)\right)}\right].$$

### 3.2. Bilayer Membrane in Strong 1-1 Electrolyte Solution (Case II)

Let a bilayer (asymmetric bipolar) membrane with a given chloride ion transport number be used (the dependence of the chloride ion transport number on current density and concentration of the external solution is known for the membranes used in this work [37,49]).

In this case, chloride ions can be transported through the bipolar membrane. The flux of chloride ions can be given by the effective transport number $T_{\mathrm{Cl}}^{\mathrm{BM}}$. As shown in [37], in dilute electrolyte solutions, depending on the thickness of the cation-exchange layer, the transport number of chloride ions for an asymmetric bipolar membrane varies within 0.2–0.5. In addition, the transport number of chloride ions depends on the concentration of the external electrolyte. In the framework of this study, we will assume that the transport number of the chloride ion is a constant value at a given current density.

Let us obtain an expression for the fluxes of chloride ions, hydrogen, and sodium ions, considering Equation (24):

(34) $$J_{\mathrm{Cl}}^{\mathrm{BM}}=\frac{iT_{\mathrm{Cl}}^{\mathrm{BM}}}{F},$$

(35) $$J_{H}^{\mathrm{BM}}=\frac{i}{F}-J_{\mathrm{Cl}}^{\mathrm{BM}}=\left(1-T_{\mathrm{Cl}}^{\mathrm{BM}}\right)\frac{i}{F},$$

(36) $$J_{\mathrm{Na}}^{C}=\frac{\left(1-T_{\mathrm{Cl}}^{\mathrm{BM}}\right)i}{F}\frac{D_{\mathrm{Na}}c_{\mathrm{Na}}^{\mathrm{dc}}}{D_{\mathrm{Na}}c_{\mathrm{Na}}^{\mathrm{dc}}+D_{H}c_{H}^{\mathrm{dc}}}.$$

The concentration of chloride ions in the desalination chamber can be found from Equation (7) taking into account Equation (34) when integrating with the condition that at τ=0 $c_{\mathrm{Cl}}^{\mathrm{dc}}=c_{\mathrm{Cl}}^{\mathrm{dc}}(0)$, and at some τ>0 $c_{\mathrm{Cl}}^{\mathrm{dc}}=c_{\mathrm{Cl}}^{\mathrm{dc}}(\tau )$:

(37) $$c_{\mathrm{Cl}}^{\mathrm{dc}}(\tau )=c_{\mathrm{Cl}}^{\mathrm{dc}}(0)-\frac{iT_{\mathrm{Cl}}^{\mathrm{BM}}}{V^{\mathrm{dc}}F}\tau .$$

From the condition of electrical neutrality, we express the concentration of hydrogen ions:

(38) $$c_{H}^{\mathrm{dc}}(\tau )=c_{H}^{\mathrm{dc}}(0)-c_{\mathrm{Na}}^{\mathrm{dc}}(\tau )+c_{\mathrm{Cl}}^{\mathrm{dc}}(\tau ).$$

After substituting $c_{H}^{\mathrm{dc}}(\tau )$ from Equation (38) into Equation (36) and solving Equation (5), we obtain:

(39) $$\left(D_{\mathrm{Na}}-D_{H}\right)c_{\mathrm{Na}}^{\mathrm{dc}}(\tau )+D_{H}\left(c_{\mathrm{Cl}}^{\mathrm{dc}}(\tau )+c_{H}^{\mathrm{dc}}(0)\right)\ln\left(c_{\mathrm{Na}}^{\mathrm{dc}}(\tau )\right)=D^{II},$$

where

(40) $$D^{II}=\left(D_{\mathrm{Na}}-D_{H}\right)c_{\mathrm{Na}}^{\mathrm{dc}}(0)+D_{H}\left(c_{\mathrm{Cl}}^{\mathrm{dc}}(\tau )+c_{H}^{\mathrm{dc}}(0)\right)\ln\left(c_{\mathrm{Na}}^{\mathrm{dc}}(0)\right)-\frac{\left(1-T_{\mathrm{Cl}}^{\mathrm{BM}}\right)iD_{\mathrm{Na}}}{V^{\mathrm{dc}}F}\tau .$$

Analytical expression for $c_{\mathrm{Na}}^{\mathrm{dc}}(\tau )$:

(41) $$c_{\mathrm{Na}}^{\mathrm{dc}}(\tau )=\frac{D_{H}\left(c_{\mathrm{Cl}}^{\mathrm{dc}}(\tau )+c_{H}^{\mathrm{dc}}(0)\right)}{D_{\mathrm{Na}}-D_{H}}W\left(z^{II}\right),$$

where

(42) $$z^{II}=\frac{\left(D_{\mathrm{Na}}-D_{H}\right)c_{\mathrm{Na}}^{\mathrm{dc}}(0)}{D_{H}\left(c_{\mathrm{Cl}}^{\mathrm{dc}}(\tau )+c_{H}^{\mathrm{dc}}(0)\right)}\exp\left[\frac{\left(D_{\mathrm{Na}}-D_{H}\right)c_{\mathrm{Na}}^{\mathrm{dc}}(0)-\left(1-T_{\mathrm{Cl}}^{\mathrm{BM}}\right)iD_{\mathrm{Na}}\tau }{V^{\mathrm{dc}}FD_{H}\left(c_{\mathrm{Cl}}^{\mathrm{dc}}(\tau )+c_{H}^{\mathrm{dc}}(0)\right)}\right].$$

### 3.3. Ideally Selective Bipolar Membrane in Solution Containing Weak Electrolyte Anions (Case III)

Let a weak electrolyte solution (for example, sodium acetate) circulate in the desalting and concentration path. In the system under consideration, chlorine anions are replaced by anions of a weak electrolyte, and a new substance is added—the molecular form of a weak electrolyte.

In the presence of weak electrolyte ions, the system of Equations (2)–(23) must be supplemented by the chemical equilibrium equation and the material balance condition (written in general form for a monobasic carboxylic acid):

(43) $$\mathrm{HA}⇄H^{+}+A^{-},\;K_{d}=\frac{\left[H^{+}\right]\left[A^{-}\right]}{\left[\mathrm{HA}\right]},$$

(44) $$c_{A}=c_{A^{-}}+c_{\mathrm{HA}}.$$

The condition of ideal selectivity of the bipolar membrane assumes that in the desalting chamber there is no loss of weak electrolyte anions due to migration, as well as weak electrolyte molecules due to diffusion through the bipolar membrane. Mathematically, this condition is expressed as:

(45) $$V^{\mathrm{dc}}(\tau )\frac{dc_{A}^{\mathrm{dc}}}{d\tau }=0.$$

At the same time, as was shown in [50], the concentration of ions and the molecular form of a weak electrolyte changes with time due to the occurrence of a chemical reaction:

(46) $$V^{\mathrm{dc}}(\tau )\frac{dc_{A^{-}}^{\mathrm{dc}}}{d\tau }=-J^{r},$$

(47) $$V^{\mathrm{dc}}(\tau )\frac{dc_{\mathrm{HA}}^{\mathrm{dc}}}{d\tau }=J^{r}.$$

In such a system, a change in the concentration of hydrogen ions in the desalination chamber will be associated with their generation on the bipolar membrane, their competitive transfer through the cation-exchange membrane, and a decrease because of the chemical reaction:

(48) $$V^{\mathrm{dc}}(\tau )\frac{dc_{H}^{\mathrm{dc}}}{d\tau }=J_{H}^{\mathrm{BM}}-J_{H}^{C}-J^{r}.$$

Electroneutrality condition:

(49) $$c_{\mathrm{Na}}^{\mathrm{dc}}(\tau )+c_{H}^{\mathrm{dc}}(\tau )=c_{A^{-}}^{\mathrm{dc}}(\tau ).$$

At the initial moment of time, no hydrogen ions generated on the bipolar membrane can reach the surface of the cation-exchange membrane, because they participate in the chemical recombination reaction with weak electrolyte anions. Under such conditions, the flow of hydrogen ions through the cation exchange membrane can be neglected, and the flow of sodium ions through the cation exchange membrane can be assumed to be a constant proportional to the current. Then the sodium ion concentration at time *τ* can be found as:

(50) $$c_{\mathrm{Na}}^{\mathrm{dc}}(\tau )=c_{\mathrm{Na}}^{\mathrm{dc}}(0)-\frac{iT_{\mathrm{Na}}^{c}}{F}\tau .$$

Using Equations (43) and (44) we obtain the expression for $c_{H}^{\mathrm{dc}}(\tau )$:

(51) $$c_{H}^{\mathrm{dc}}(\tau )=-\frac{1}{2}\left(K_{d}+c_{\mathrm{Na}}^{\mathrm{dc}}(\tau )-\sqrt{{\left(c_{\mathrm{Na}}^{\mathrm{dc}}(\tau )-K_{d}\right)}^{2}+4K_{d}c_{A}}\right).$$

Knowing the initial concentrations of ions in the solution and the current density, one can directly calculate the concentration of sodium ions using Equation (50), and then find the concentration of hydrogen ions (Equation (51)). The anion concentration can be found using the following equation:

(52) $$c_{A^{-}}=\frac{K_{d}c_{A}}{c_{H}+K_{d}},$$

the concentration of the molecular form can be found from the chemical equilibrium equation (Equation (43)).

### 3.4. Ideally Selective Bipolar Membrane in a Solution Containing a Strong and a Weak Electrolyte (Case IV)

Let a solution containing ions of strong and weak electrolytes circulate in the desalination and concentration path (for example, a mixture of sodium chloride and sodium acetate).

This case differs from the previous one in that one more term appears in the electrical neutrality equation:

(53) $$c_{\mathrm{Na}}^{\mathrm{dc}}(\tau )+c_{H}^{\mathrm{dc}}(\tau )=c_{A^{-}}^{\mathrm{dc}}(\tau )+c_{\mathrm{Cl}}^{\mathrm{dc}}(\tau ).$$

Considering that the bipolar membrane is assumed to be ideally selective, the changes in the concentration of chloride ions in the desalination chamber can be equated to zero, which means that $c_{\mathrm{Cl}}^{\mathrm{dc}}(\tau )=c_{\mathrm{Cl}}^{\mathrm{dc}}(0)$. Substituting the last expression into Equation (53) and solving the resulting quadratic equation for the concentration of hydrogen ions, we obtain:

(54) $$c_{H}^{\mathrm{dc}}(\tau )=-\frac{1}{2}\left(\left(K_{d}+c_{\mathrm{Na}}^{\mathrm{dc}}(\tau )-c_{\mathrm{Cl}}^{\mathrm{dc}}(0)\right)-\sqrt{{\left(K_{d}+c_{\mathrm{Na}}^{\mathrm{dc}}(\tau )-c_{\mathrm{Cl}}^{\mathrm{dc}}(0)\right)}^{2}-4K_{d}\left(c_{\mathrm{Na}}^{\mathrm{dc}}(\tau )-c_{A}-K_{d}\right)}\right).$$

The concentration of sodium ions, as well as in the previous case, can be found by assuming the flux of sodium ions to be constant in time, using Equation (50). This condition is well satisfied when the degree of conversion of a weak electrolyte is less than 90%. After calculating the concentration of hydrogen ions according to Equation (54), the further calculation algorithm is similar to case III.

### 3.5. Bilayer Membrane in Solution Containing Strong and Weak Electrolyte (Case V)

In addition to the previous case, let a bilayer (asymmetric bipolar) membrane with a given anion transport number be used.

In this system, as well as in the previous two cases, we will neglect the transfer of anions of a weak electrolyte through a bilayer membrane, as well as the diffusion transfer of the molecular form of a weak electrolyte. This is possible when the reaction of protonation of anions of a weak electrolyte occurs near the surface of a bilayer membrane, the diffusion transfer of the molecular form of a weak electrolyte is directed into the solution [83]. Due to the occurrence of a chemical reaction, free hydrogen ions do not accumulate in the solution for a long time, and the cation flux can be assumed constant over time. The flux of chloride ions is proportional to the effective transport number (Equation (37)).

Considering the above assumptions, we obtain an equation similar to Equation (54):

(55) $$c_{H}^{\mathrm{dc}}(\tau )=-\frac{1}{2}\left(\left(K_{d}+c_{\mathrm{Na}}^{\mathrm{dc}}(\tau )-c_{\mathrm{Cl}}^{\mathrm{dc}}(\tau )\right)-\sqrt{{\left(K_{d}+c_{\mathrm{Na}}^{\mathrm{dc}}(\tau )-c_{\mathrm{Cl}}^{\mathrm{dc}}(\tau )\right)}^{2}-4K_{d}\left(c_{\mathrm{Na}}^{\mathrm{dc}}(\tau )-c_{A}-K_{d}\right)}\right).$$

After calculating the concentration of hydrogen ions according to Equation (55), the further calculation algorithm is similar to case III.

## 4. Results and Discussion

### 4.1. The Results of Adjusting the pH of the NaCl Solution with Simultaneous Desalting in Circulation Mode

The studies were carried out on a solution of sodium chloride with a concentration of 0.02 M. During the experiment, the kinetic dependences of the salt concentration and pH, as well as the volume of the solution in the desalting compartment (including solution in the pipes, inner volume of the ED module and solution tank itself) of the desalination and concentration compartments, were studied. The experiment to adjust the pH of the sodium chloride solution was carried out until the concentration of sodium chloride in the desalination chamber reached 0.01 M.

The tests showed that in the framework of the experiment, there was no significant change in the volume of the solution both in the desalting tract and in the concentration tract.

**Figure 4:**
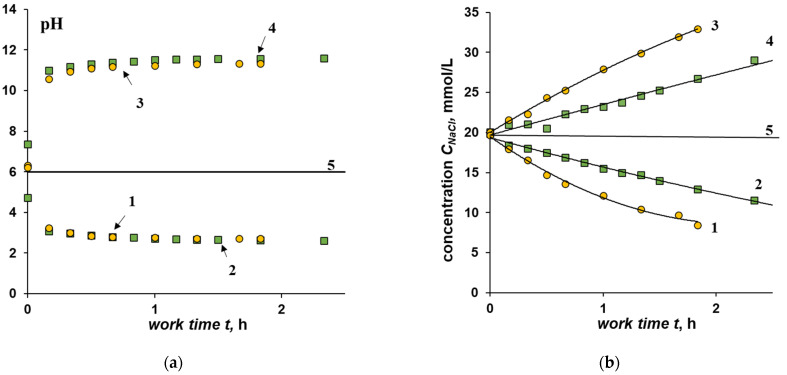
Dependence of the pH index (**a**) and the concentration of sodium chloride (**b**) in the desalination (1, 2) and concentration (3, 4) compartments on the experiment time at a current density of 10 mA/cm^2^. Bilayer membranes: 1, 3—BM-ac-2K, 2, 4—BM-a-30. Line 5—initial concentration and pH.

Figure 4 shows the kinetic curves of changes in pH and sodium chloride concentration in the desalting and concentration chambers for electrodialyzers with bilayer BM-a-30 and BM-ac-2K at a current density of 10 mA/cm^2^.

At a constant current density, the required solution concentration in the desalination chamber is reached faster for a membrane with a catalyst (BM-ac-2K), while the final pH value both in the desalination channel and in the concentration, channel does not depend on the activity of the bilayer membrane in the water dissociation reaction (Figure 4).

**Figure 5:**
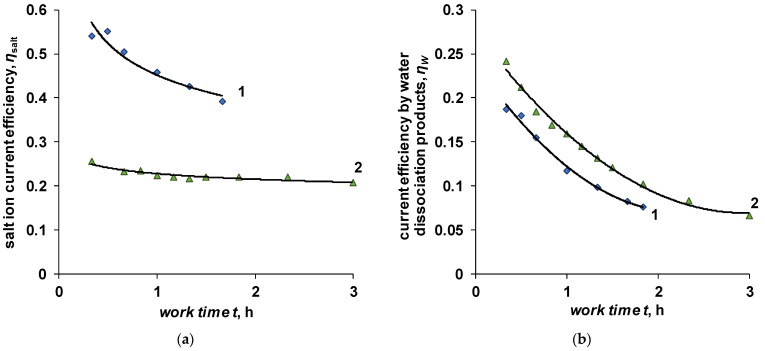
Dependence of the current efficiency on salt ions (**a**) and water dissociation products (**b**) on the experiment time at a current density of 10 mA/cm^2^. Bilayer membrane: 1—BM-ac-2K, 2—BM-a-30.

Faster desalination of the solution when using the BM-ac-2K bilayer membrane is due to the higher limiting current, which is associated with the presence of catalyst particles in the cation-exchange layer, the selectivity of which to co-ions is lower compared to sulfonated polytetrafluoroethylene [37]. An assessment of the integral current efficiency, carried out for a current density of 10 mA/cm^2^, shows that the salt ions’ current efficiency for BM-ac-2K membranes is, on average, twice as high as compared to the BM-a-30 membrane. At the same time, the current efficiency in terms of water dissociation products is only 20% lower (Figure 5).

As applied to processes that require simultaneous pH adjustment and solution desalination, the high salt permeability of a bilayer membrane with a catalyst for the water dissociation reaction has a positive effect on the process characteristics.

A comparison of the calculated curves for cases I and II with the experimental results is shown in Figure 5. In the calculations, a current density of 10 mA/cm^2^ was used, which slightly exceeds the limiting current density on the Ralex CMH cation-exchange membrane found experimentally from the current-voltage characteristics of the membrane in the membrane package of the electrodialyzer (9.8 mA/cm^2^).

**Figure 6:**
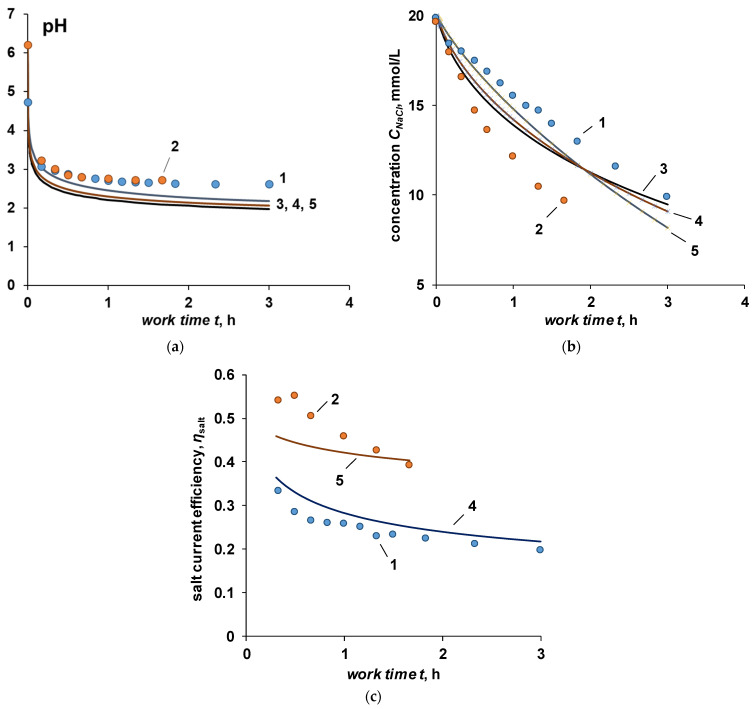
Experimental and calculated dependences of pH (**a**), concentration (**b**), and current efficiency (**c**) for sodium chloride on the time of the experiment at a current density of 10 mA/cm^2^. The points are the experiment, the lines are the results of the calculation. 1—bilayer membrane BM-a-30, 2—bilayer membrane BM-ac-2K, 3—calculation for case I (ideally selective BPM), 4—calculation for case II ($T_{\mathrm{Cl}}^{\mathrm{BM}}$ = 0.22), 5—calculation for case II ($T_{\mathrm{Cl}}^{\mathrm{BM}}$ = 0.53).

As can be seen from the data shown in Figure 6a, the pH of the solution in the desalination compartment quickly decreases to a value of about 3.5 in the first minutes of operation of the apparatus, after which the decrease slows down. During the first 20 min, the pH drops to 2.5 and then changes very slowly, due to the competitive transfer of the hydrogen ion through the cation exchange membrane. All calculated curves give final pH values lower than the value observed in the experiment. A possible explanation for this effect is the violation of condition (10) in the real process. The transfer of hydroxyl ions, as co-ions, through the cation-exchange membrane is possible, if they are in excess in the concentration chamber.

The rate of change in the pH value, as well as its final value, are practically independent of the value of the chloride ion transfer number through the bilayer membrane (in fact, they do not depend on the efficiency of the bilayer membrane in the transfer of water dissociation reaction products), which was also observed in the experiment. The final pH value of the solution in the desalination chamber is determined by the specific permeability coefficient of the cation-exchange membrane in the Na^+^/H^+^ pair. At an overlimiting current regime, the value of this coefficient becomes constant and is determined by the ratio of the charges of competing ions and their diffusion coefficients in solution. Thus, when working with current densities exceeding the limit value on the cation exchange membrane, the final pH value is also independent of the cation exchange membrane used.

The calculation of the dependence of the salt concentration in the desalination channel on the duration of the experiment (Figure 6b) and the current efficiency in terms of salt ions (Figure 6c) shows a good agreement between the experimental and calculated data for a bilayer membrane without a catalyst (calculation for case II, $T_{\mathrm{Cl}}^{\mathrm{BM}}$ = 0.22). For a membrane with a catalyst (calculation for case II, $T_{\mathrm{Cl}}^{\mathrm{BM}}$ = 0.53), there is a significant discrepancy between the experimental and calculated curves, which is especially pronounced on the curve of the dependence of the salt ion current efficiency on time. In both cases, the value of the transport number of chloride ions through the bilayer membrane was taken from the experimental data given in [37].

### 4.2. Conversion of Sodium Acetate to Acetic Acid

The process of obtaining a weak organic acid from its salt is closest to the conditions described by case III (an ideally selective bipolar membrane in a solution containing anions of a weak electrolyte). There are many examples of using this process to obtain acetic, citric, lactic, succinic, and other organic acids [25]. As a rule, the conversion process is carried out from sufficiently concentrated solutions, therefore, in this experiment, 0.5 M sodium acetate was used as the initial solution. Experimental data shown in this subsection was obtained using laboratory level MB-2m bipolar membrane (heterogeneous bipolar membrane with low water-splitting potential and high selectivity) [79].

**Figure 7:**
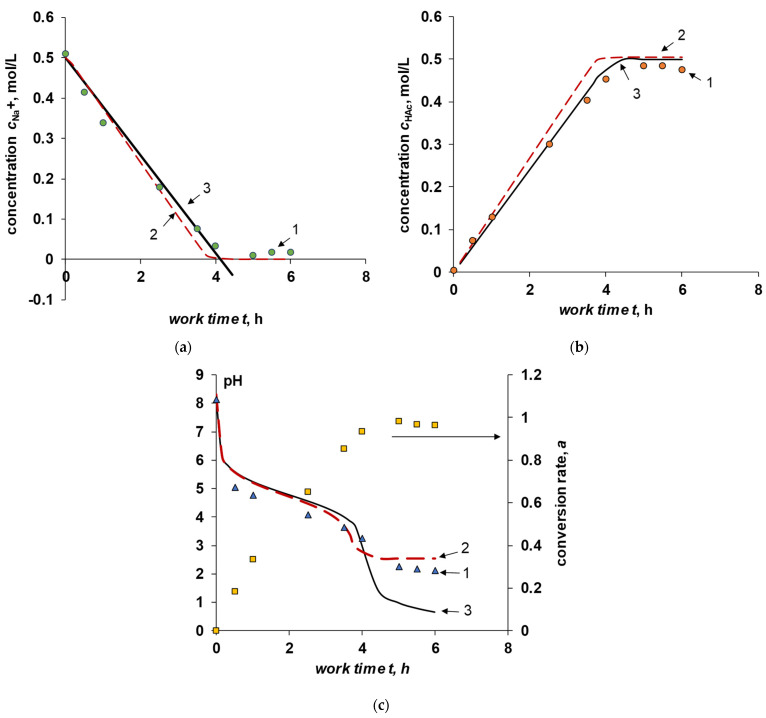
Experimental and calculated dependences of sodium acetate (**a**), acetic acid (**b**), and pH of the sodium acetate solution (**c**) on the time of the experiment at a current density of 20 mA/cm^2^. 1—experimental data, 2—calculation for case III, 3—calculation for case III taking into account proton transport through the cation-exchange membrane, 4—sodium acetate conversion rate.

The kinetic dependences of the pH and the concentrations of sodium acetate and acetic acid are shown in the Figure 7.

It can be seen from the figure that the assumptions about the linear nature of the removal of sodium ions (and, in general, salt) made for case III are well fulfilled up to a degree of conversion of 0.93. After that, in the experiment, the concentration of sodium acetate and acetic acid reaches a plateau, accompanied by a decrease and reaching a plateau in the pH value. When the degree of conversion is higher than 0.93, a small amount of unbound acetate ions remains in the solution, resulting in an increase in the concentration of hydrogen ions generated by the bipolar membrane. The appearance of “free” protons leads to the competitive transfer of hydrogen and sodium ions through the cation exchange membrane. As a result, it becomes necessary to consider condition (14). The calculation results considering the competitive transfer of protons and sodium ions are shown in the Figure 7 by a dashed line (curves 3). These show that considering the competitive transfer leads to a more accurate prediction of the concentration of ionic components and pH. In fact, the beginning of the competitive transfer of sodium and hydrogen ions through the cation-exchange membrane can be considered as a deviation of the number of transferred sodium ions through the cation-exchange membrane from unity (to calculate curves 2 in Figure 7, the value $T_{\mathrm{Na}}^{C}=1$ was used). Hydrogen ions become the main charge carrier through the cation-exchange membrane at high degrees of conversion.

The accumulation of molecular acetic acid also leads to an increase in the diffusion transfer of acid through the bipolar membrane, resulting in a slight decrease in the concentration of molecular acetic acid at the end of the experiment.

The equation for calculating the concentration of salt ions for case III, considering competitive transfer, is given in Table A1, Appendix A.

### 4.3. Adjusting pH in a Mixture of Strong and Weak Electrolytes

The separation of a mixture of strong and weak electrolyte salts was carried out using a Ralex CM/BM-ac-2K membrane pair. A model solution, a mixture of 0.02 M sodium chloride and 0.02 M sodium acetate, was used for the studies. During the research, kinetic curves of the dependence of pH, electrical conductivity, and concentration of solution components on the time of the process were obtained. The BM-ac-2K bilayer membrane at high current densities provides efficient generation of H^+^/OH^−^ ions and can be used in dilute solutions as a replacement for the bipolar one. At low or “medium” current densities, this membrane provides a sufficiently high transfer of salt ions contained in the solution (transport numbers for chloride ions lie in the range of 0.3–0.7 [37,50]).

**Figure 8:**
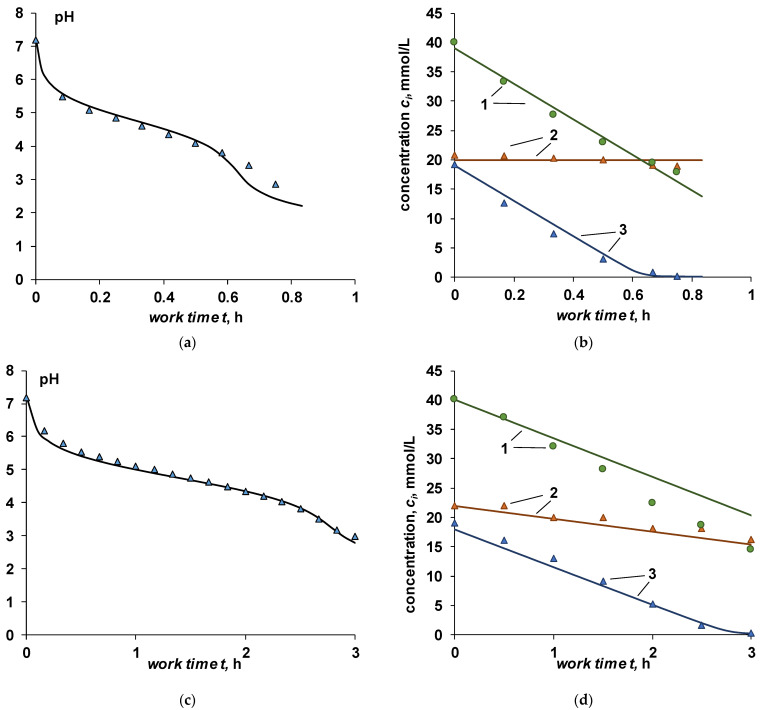
Dependence of the pH (**a**,**c**) and the concentration of ionic components (**b**,**d**) on time. Current density: (**a**,**b**)—33 mA/cm^2^, (**c**,**d**)—10 mA/cm^2^. 1—Na^+^, 2—Cl^–^, 3—CH3COO^–^. Points—experimental data, lines—calculation for case IV (**b**) and case V (**d**).

The kinetic curves of the pH dependence for a mixture of strong and weak electrolytes do not fundamentally differ from the case of the conversion of a salt of a weak electrolyte into an acid (case III) (Figure 8a,c). From the kinetic curves of the concentration of the ionic components of the solution, it follows that the concentration of the cation (sodium ion) decreases almost linearly throughout the entire process, which indicates the absence of a competitive transfer of cations through the cation-exchange membrane. The reason for this is the binding of protons to acetate ions. The experimental data confirm the assumptions made when deriving the equations for cases IV and V. The concentration of chloride ions at high current density (Figure 8b) does not change during the experiment, which corresponds to the condition of ideal selectivity of the bilayer membrane. At a lower current density during the experiment, there is a slight decrease in the concentration of chloride ions, as well as the total content of acetate (both forms).

Comparison of calculation results and experimental data on pH and ion concentration in the desalination chamber using Equations (42)–(44) (case IV) is shown in Figure 8a,b. Despite the fact that the BM-ac-2K bilayer membrane does not fully meet the requirement of ideal selectivity laid down for case IV, the agreement between the calculated curves and experimental data is quite good.

In the case when the permeability of the bilayer membrane for anions in solution is taken into account, there is also a good agreement between the experiment and calculations, assuming that the transfer numbers of sodium ions through the cation-exchange membrane and chloride ions through the bilayer membrane are chosen correctly (Figure 8c,d). In the calculations shown in Figure 8c,d, the following values were used $T_{\mathrm{Na}}^{C}=0.65$, $T_{\mathrm{Cl}}^{\mathrm{BM}}=0.23$. The value of the transport number of chloride ions was taken from experimental data for a current density of 10 mA/cm^2^ [50]. The value of the transfer number of the sodium ion is calculated as the sum of the transfer numbers of chloride ions and hydrogen ions.

### 4.4. The Possibility of Using the Results for the Calculation of Electrodialyzers-Synthesizers

The proposed kinetic model can be used for the initial calculation of bipolar electrodialyzers designed to adjust pH. This model makes it possible to find the optimal length of a membrane channel formed by a bipolar membrane and a cation exchange membrane, depending on the pH to be reached at the outlet of the desalination or concentration channel. For calculations, it is necessary to know the composition of the solution and the effective numbers of ion transfer through monopolar membranes in this solution. Because solutions that require only slight pH adjustments tend to have low salinity, cases of ideal bipolar membrane selectivity (cases I, III and IV) can be used for calculations.

A simple kinetic model can be used to calculate the concentrations of all components present in the alkaline and acid chambers of an electrodialysis cell with a channel length of up to 40 cm. When the length of the membrane channel is more than 40 cm, it is necessary to use more complex mathematical approaches, for example, the numerical solution of the model proposed in [78,84]. The need for a numerical calculation is associated with the nonlinear distribution of the current density along the length of the channel, which occurs due to the large difference in the concentration of the components at its inlet and outlet. The principle of modeling in long channels consists of dividing the length of the membrane channel into a sufficiently large number of sections of smaller length, located perpendicular to the direction of the solution flow. The calculation is carried out in such a way that the output calculated data on the previous section are the input data for the next section (compartmentation method [85,86]). The method allows layer-by-layer calculation of the composition of the solution as it passes through the channel and thus finds the local value of ion concentrations and pH. The developed model makes it possible to obtain the dependence of the pH of the solution on the length of the electrodialyzer channel, which is especially important when predicting the mass transfer characteristics of industrial devices.

Various approaches can be used to consider the uneven distribution of the current density along the channel length. Tanaka [87,88,89] used a third degree polynomial to describe the current density distribution along the length of the electrodialyzer channel.

When operating in a potentiostatic mode (a necessary condition for applying the compartmentation method), the decrease in current density along the channel length can be described by the equation:

(56) $$i=\frac{F^{2}}{RT}\frac{U-\frac{RT}{F}\ln\left(\frac{c_{H}^{\mathrm{cc}}}{c_{H}^{\mathrm{dc}}}\right)}{\left(\frac{1}{\sum z_{i}^{2}D_{i}c_{i}^{\mathrm{dc}}}+\frac{1}{\sum z_{i}^{2}D_{i}c_{i}^{\mathrm{cc}}}\right)h},$$

where *U* is the potential drop on solutions in desalinating and concentration compartments, *z_i_* and *D_i_* are the charge and diffusion coefficients of ion of *i*-type.

The above equation does not consider the resistance of the cation-exchange membrane and the monopolar layers of the bipolar membrane, because in dilute solutions their electrical conductivity is several times higher than the electrical conductivity of the solution. The potential drop at the bipolar boundary caused by the water dissociation reaction, which is non-ohmic in nature, is also not considered. This potential drop must be included both in the total potential drop on the channel and subtracted from it. As the calculations are made for the case of an ideally selective bipolar membrane, it can be taken equal to the thermodynamic potential of water dissociation. In the first approximation, it does not depend on the composition of the solution but is determined by the concentration of hydrogen ions in the cation-exchange and anion-exchange layers (in fact, the exchange capacity of each of the layers), then its change along the channel length can be neglected and considered constant.

**Figure 9:**
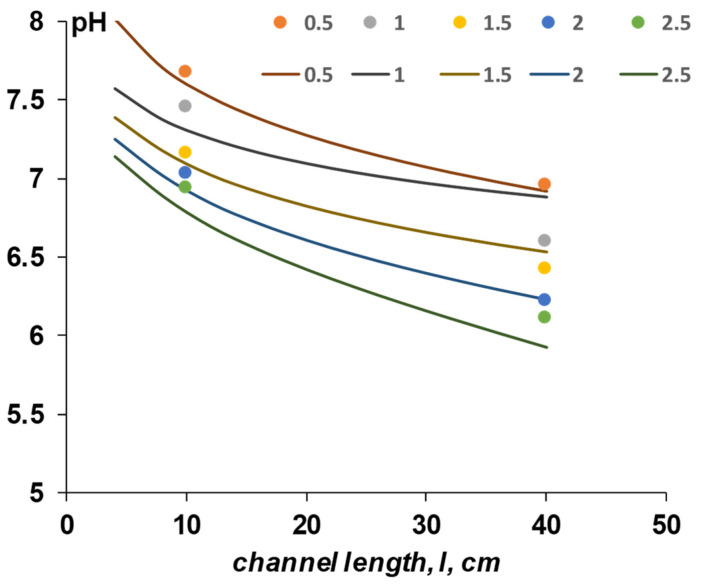
Dependence of the pH of the solution at the outlet of the acidic chambers of the electrodialyzer with bipolar membranes on the channel length at different current densities Points are experimental values, curves are calculation by the model/Numbers in the legend represent the current density in mA/cm^2^.

As a result of solving the mathematical model, a dataset of the pH dependence of the solution in the acid and alkaline chambers of the electrodialyzer is obtained depending on the current density and the length of the membrane channel (Figure 9). Softened tap water from the city of Krasnodar was used as a solution. The concentration of main ionic components of the tap water are given in the Table A2, Appendix B. The process of electrodialysis of softened tap water is complicated by chemical reactions involving single- and double-charged forms of carbonic acid when the pH changes in the acid and alkaline chambers. Because of those reactions involved, we used case IV for the calculation. A solution velocity was set to 2.5 cm/s (as in experiment). We used the same experimental setup described in Section 4.1 to measure the pH at the outlet for the channel length 10 cm. A bigger electrodialysis unit was used to measure the pH at the outlet for the channel length 40 cm. The properties of the later electrodialysis unit can be found in the Table A3, Appendix C.

Figure 9 shows that all the calculated curves describe the experimental data qualitatively well. The best agreement between experimental data and calculated curves is observed for current densities of 0.5, 1.5, 2.0 mA/cm^2^. For the highest current density studied (2.5 mA/cm^2^), the calculated pH values are lower than the experimental data.

Knowing the working dimensions of one chamber of the electrodialyzer, the volumetric flow rate of the solution, and the linear velocity of the solution flow through the electrodialyzer chamber, we can find the number of paired chambers that are required to adjust the pH to a given value:

(57) $$n=\frac{Q}{Vwhg},$$

where *n* is the number of elementary cells, *Q* is the volume flow rate, m^3^/h, *V* is the solution linear velocity, m/s, *w* is the width of the membrane channel, m, *h* is the intermembrane distance (the thickness of the separator), m, *g* is the separator porosity (0.85 for separators used in present work).

For calculations, it is proposed to use the following algorithm:

- set the composition of the solution,
- set the required linear velocity of the solution flow through the chamber,
- calculate possible options for adjusting pH for various channel lengths and current densities and choose the most appropriate option for a particular case,
- choose the closest standard electrodialyzer in terms of dimensions,
- calculate the required amount of membrane pairs, add 10% to the calculated amount,
- if the obtained number of membrane pairs exceeds the recommended maximum number of membrane pairs for this device, repeat the calculation for the next electrodialysis module (Table 3).

## 5. Conclusions

In this work, we have proposed a kinetic model of the process of bipolar electrodialysis with a two-chamber unit cell formed by a bilayer (bipolar or asymmetric bipolar) and cation-exchange membrane. In contrast to the known mathematical models, the proposed model allows describing the process of electrodialysis adjustment of the pH of solutions of strong electrolytes, the conversion of a salt of a weak acid, and the adjustment of the pH of a mixture of strong and weak electrolytes. The model can consider the non-ideal selectivity of the bilayer membrane, as well as the competitive transfer of cations (hydrogen and sodium ions) through the cation-exchange membrane. If necessary, it is possible to consider changes in the volume of solutions in the desalination and concentration compartments. The model can be used to both describe processes occurring in a cycle and in a one-pass through mode.

A number of approximations were used to obtain analytical expressions that describe the kinetic dependences of pH and concentration of ionic components in the desalination (acidification) compartment. The assumptions are the constancy of the volume of solutions in the compartments, the over-limiting current mode on the cation-exchange membrane, the transport numbers through bilayer membrane are known, the absence of transfer of co-ions through cation-exchange membranes. Similar expressions can be obtained for the concentration (alkalinization) compartment. A good qualitative and, in some cases, quantitative agreement between the calculations and experimental results is shown.

The model can be used to predict the performance of small bipolar membrane electrodialysis modules designed for pH adjustment processes. Of greatest interest is the use of the model to describe the processes of adjusting the pH of mixed solutions of strong and weak electrolytes (adjusting the pH of fruit juices, softened water, separation of amino acids and salts).

## Figures and Tables

**Table 1:** Physicochemical properties of the Ralex CM-Pes membranes.

Ion-exchange resin	Lewatit S100 (LANXESS, Berlin, Germany)
Inert binder	LDPE LD605BA (ExxonMobil Chemical, Houston, TX, USA)
Reinforcing mesh	Ulester 32S (SILK & PROGRESS, Brněnec, Czech Republic)
Functional groups	-SO_3_^−^
Ion-exchange capacity, mmol/g-swollen	1.10
Wet thickness, microns	690
Water content, %	44.5
Surface resistance ^1^, Ohm•cm^2^	8.2 ± 0.2

^1^ measured in 0.5 M NaCl.

**Table 2:** Physicochemical properties of the bilayer membranes.

Membrane	MB-2m	BM-a-30	BM-ac-2K
Polymeric matrix	Polystyrene divinylbenzene	Polystyrene divinylbenzene ^1^/Polytetrafluorideethylene ^2^	Polystyrene divinylbenzene ^1^/Polytetrafluorideethylene ^2^
Wet thickness, mm	0.66 ± 0.1	0.48 ± 0.01	0.48 ± 0.01
Ion-exchange capacity, mmol/g-swollen:			
Cation-exchange layer	1.4 ± 0.2	0.8 ± 0.05	0.8 ± 0.05
Anion-exchange layer	1.4 ± 0.2	1.1 ± 0.2	1.1 ± 0.2
Ionic group:			
Cation-exchange layer	–SO_3_^–^	–SO_3_^–^	–SO_3_^–^
Anion-exchange layer	–N^+^(CH_3_)_3_	–N^+^(CH_3_)_3_	–N^+^(CH_3_)_3_
Water-splitting catalyst	ion-polymer containing phosphoric acid ionic groups	-	cation exchange resin KF-1
Potential drop, *V* (*i* = 1 A/dm^2^ in 0.01 M NaCl) ^3^	1.0 ± 0.1	8 ± 0.4	3.8 ± 0.1

^1^ Anion-exchange layer; ^2^ Cation-exchange layer; ^3^ Conditions close to the experimental.

**Table 3:** Electrodialysis modules.

Electrodialysis Module	External Dimensions of the Membrane, cm	Active Surface of the Membrane, cm^2^	Effective Area of the Membrane	Number of Elementary Cells	Productivity of the Device, m^3^/h	Refs.
**Lab EDS**	7.5 × 25	5 × 20	0.53	5–25	0.02–0.1	[79,90]
EDS-Y *	15 × 52	10 × 40	0.51	10–50	0.05–1	[86]
**EDS-m**	40 × 100	33 × 70	0.57	25–100	1–5	[91]
**EDS-10**	44 × 120	33 × 90	0.55	50–200	2–10	-

* same dimensions as EDC-Y described in ref. [86].

## Data Availability

Not applicable.

## List of Symbols and Abbreviations

*Subscripts and superscripts:*		
Upper indexes “dc” and “cc” refer to the parameters in the desalination and concentration compartments.		
Upper indexes “BM” and “C” refer to the transfer of ions through bilayer and cation-exchange membrane.		
Lower indexes “Na”, “Cl”, “H”, “OH” refer to the sodium, chloride, hydrogen, hydroxyl ions.		
Lower indexes “A^–^” and “A” refer to the ionic form of weak electrolyte and to the overall concentration of all forms of weak electrolyte.		
*Abbreviations:*		
ED	electrodialysis	
BMED	bipolar membranes	
BPM	bipolar membrane	
BM	bilayer membrane	
*Greek letters:*		
Parameter	Description	Dimensions
τ	time	s
*English letters:*		
Parameter	Description	Dimensions
*c*	concentration	mol/L
*D*	diffusion coefficient	cm^2^/s
*F*	Faraday’s constant	A·s/mol
*g*	separator porosity	
*h*	intermembrane distance	cm
*i*	current density	A/dm^2^
*J*	ion flux	mol/s
*K_d_*	weak electrolyte dissociation constant	
*K_w_*	ionic product of water	
*n*	number of elementary cells	
*P_Na/H_*	specific permeability coefficient	
*Q*	solution volume flow rate	m^3^/h
*r*	solute flux proportionality factor	
*R*	universal gas constant	J/(K·mol)
*S*	membrane area	dm^2^
*T*	absolute temperature	K
*T_i_*	ion effective transport number	
*U*	potential drop	V
*v*	solution linear velocity	m/s
*V*	solution volume	L
*w*	width of the membrane channel	m
*W(z)*	Lambert W-function of z argument	
*z_i_*	charge of ion of i-type	

## Appendix A

**Table A1:** Summary of equations used for calculations (for desalting (acidification) compartment).

Case	Ion	Equation
Ideally selective bipolar membrane in strong 1-1 electrolyte solution (case **I**)	Na^+^	$c_{\mathrm{Na}}^{\mathrm{dc}}(\tau )=\frac{D_{H}\left(c_{\mathrm{Na}}^{\mathrm{dc}}(0)+c_{H}^{\mathrm{dc}}(0)\right)}{D_{\mathrm{Na}}-D_{H}}W\left(z^{I}\right)$ $,\;z^{I}=\frac{D_{\mathrm{Na}}-D_{H}}{D_{H}\left(c_{\mathrm{Na}}^{\mathrm{dc}}(0)+c_{H}^{\mathrm{dc}}(0)\right)}\exp[\frac{D^{I}}{D_{H}\left(c_{\mathrm{Na}}^{\mathrm{dc}}(0)+c_{H}^{\mathrm{dc}}(0)\right)}],$ $\;D^{I}=\left(D_{\mathrm{Na}}-D_{H}\right)c_{\mathrm{Na}}^{\mathrm{dc}}(0)+D_{H}\left(c_{\mathrm{Na}}^{\mathrm{dc}}(0)+c_{H}^{\mathrm{dc}}(0)\right)\ln\left(c_{\mathrm{Na}}^{\mathrm{dc}}(0)\right)-\frac{iD_{\mathrm{Na}}}{V^{\mathrm{dc}}F}\tau$
	H^+^	$c_{H}^{\mathrm{dc}}(\tau )=c_{\mathrm{Na}}^{\mathrm{dc}}(0)+c_{H}^{\mathrm{dc}}(0)-c_{\mathrm{Na}}^{\mathrm{dc}}(\tau )$
	Cl^−^	Remains constant
Bilayer membrane in strong 1-1 electrolyte solution (case **II**)	Na^+^	$c_{\mathrm{Na}}^{\mathrm{dc}}(\tau )=\frac{D_{H}\left(c_{\mathrm{Cl}}^{\mathrm{dc}}(\tau )+c_{H}^{\mathrm{dc}}(0)\right)}{D_{\mathrm{Na}}-D_{H}}W(z^{II}),$ $z^{II}=\frac{\left(D_{\mathrm{Na}}-D_{H}\right)c_{\mathrm{Na}}^{\mathrm{dc}}(0)}{D_{H}\left(c_{\mathrm{Cl}}^{\mathrm{dc}}(\tau )+c_{H}^{\mathrm{dc}}(0)\right)}\exp\left[\frac{\left(D_{\mathrm{Na}}-D_{H}\right)c_{\mathrm{Na}}^{\mathrm{dc}}(0)-\left(1-T_{\mathrm{Cl}}^{\mathrm{BM}}\right)iD_{\mathrm{Na}}\tau }{V^{\mathrm{dc}}FD_{H}\left(c_{\mathrm{Cl}}^{\mathrm{dc}}(\tau )+c_{H}^{\mathrm{dc}}(0)\right)}\right]$, $D^{II}=\left(D_{\mathrm{Na}}-D_{H}\right)c_{\mathrm{Na}}^{\mathrm{dc}}(0)+D_{H}\left(c_{\mathrm{Cl}}^{\mathrm{dc}}(\tau )+c_{H}^{\mathrm{dc}}(0)\right)\ln\left(c_{\mathrm{Na}}^{\mathrm{dc}}(0)\right)-\frac{\left(1-T_{\mathrm{Cl}}^{\mathrm{BM}}\right)iD_{\mathrm{Na}}}{V^{\mathrm{dc}}F}\tau$
	H^+^	$c_{H}^{\mathrm{dc}}(\tau )=c_{H}^{\mathrm{dc}}(0)-c_{\mathrm{Na}}^{\mathrm{dc}}(\tau )+c_{\mathrm{Cl}}^{\mathrm{dc}}(\tau )$
	Cl^−^	$c_{\mathrm{Cl}}^{\mathrm{dc}}(\tau )=c_{\mathrm{Cl}}^{\mathrm{dc}}(0)-\frac{iT_{\mathrm{Cl}}^{\mathrm{BM}}}{V^{\mathrm{dc}}F}\tau$
Ideally selective bipolar membrane in solution containing weak electrolyte anions (case **III**)	^1^ Na^+^	$c_{\mathrm{Na}}^{\mathrm{dc}}(\tau )=c_{\mathrm{Na}}^{\mathrm{dc}}(0)-\frac{iT_{\mathrm{Na}}^{c}}{F}\tau$
	^2^ Na^+^	$\begin{array}{l} \left(2D_{\mathrm{Na}}-D_{H}\right)\left(c_{\mathrm{Na}}^{\mathrm{dc}}(\tau )-c_{\mathrm{Na}}^{\mathrm{dc}}(0)\right)-D_{H}K_{d}\ln\frac{c_{\mathrm{Na}}^{\mathrm{dc}}(\tau )}{c_{\mathrm{Na}}^{\mathrm{dc}}(0)}-D_{H}A\tanh^{-1}\left(\frac{B+K_{d}f^{\prime }}{AC^{\prime }}\right)+D_{H}C^{\prime }+\\ +D_{H}K_{d}\tanh^{-1}\left(\frac{f^{\prime }}{C^{\prime }}\right)=-\frac{2iD_{\mathrm{Na}}}{V^{\mathrm{dc}}F}\tau +\theta \end{array}$ $\theta =-D_{H}[A\tanh^{-1}\left(\frac{B+K_{d}f}{AC}\right)-C-K_{d}\tanh^{-1}\left(\frac{f}{C}\right)],$ $A=\sqrt{B+K_{d}{}^{2}}$ $,\;B=4K_{d}c_{A}$ $,\;C=\sqrt{B+f^{2}}$ $,\;f=c_{\mathrm{Na}}^{\mathrm{dc}}(0)-K_{d}$ $,\;C^{\prime }=\sqrt{B+{f^{\prime }}^{2}}$ $,\;f^{\prime }=c_{\mathrm{Na}}^{\mathrm{dc}}(\tau )-K_{d}$
	H^+^	$c_{H}^{\mathrm{dc}}(\tau )=-\frac{1}{2}\left(K_{d}+c_{\mathrm{Na}}^{\mathrm{dc}}(\tau )-\sqrt{{\left(c_{\mathrm{Na}}^{\mathrm{dc}}(\tau )-K_{d}\right)}^{2}+4K_{d}c_{A}}\right)$
	A^–^	$c_{A^{-}}=\frac{K_{d}c_{A}}{c_{H}+K_{d}}$
	Cl^−^	Remains constant
	HA	$c_{\mathrm{HA}}=c_{A}-c_{A^{-}}$
Ideally selective bipolar membrane in a solution containing a strong and a weak electrolyte (case **IV**)	Na^+^	$c_{\mathrm{Na}}^{\mathrm{dc}}(\tau )=c_{\mathrm{Na}}^{\mathrm{dc}}(0)-\frac{iT_{\mathrm{Na}}^{c}}{F}\tau$
	H^+^	$c_{H}^{\mathrm{dc}}(\tau )=-\frac{1}{2}\left(\left(K_{d}+c_{\mathrm{Na}}^{\mathrm{dc}}(\tau )-c_{\mathrm{Cl}}^{\mathrm{dc}}(0)\right)-\sqrt{{\left(K_{d}+c_{\mathrm{Na}}^{\mathrm{dc}}(\tau )-c_{\mathrm{Cl}}^{\mathrm{dc}}(0)\right)}^{2}-4K_{d}\left(c_{\mathrm{Na}}^{\mathrm{dc}}(\tau )-c_{A}-K_{d}\right)}\right)$
	A^–^	$c_{A^{-}}=\frac{K_{d}c_{A}}{c_{H}+K_{d}}$
	Cl^−^	Remains constant
	HA	$c_{\mathrm{HA}}=c_{A}-c_{A^{-}}$
Bilayer membrane in solution containing strong and weak electrolyte (case **V**)	Na^+^	$c_{\mathrm{Na}}^{\mathrm{dc}}(\tau )=c_{\mathrm{Na}}^{\mathrm{dc}}(0)-\frac{iT_{\mathrm{Na}}^{c}}{F}\tau$
	H^+^	$c_{H}^{\mathrm{dc}}(\tau )=-\frac{1}{2}\left(\left(K_{d}+c_{\mathrm{Na}}^{\mathrm{dc}}(\tau )-c_{\mathrm{Cl}}^{\mathrm{dc}}(\tau )\right)-\sqrt{{\left(K_{d}+c_{\mathrm{Na}}^{\mathrm{dc}}(\tau )-c_{\mathrm{Cl}}^{\mathrm{dc}}(\tau )\right)}^{2}-4K_{d}\left(c_{\mathrm{Na}}^{\mathrm{dc}}(\tau )-c_{A}-K_{d}\right)}\right)$
	A^–^	$c_{A^{-}}=\frac{K_{d}c_{A}}{c_{H}+K_{d}}$
	Cl^−^	$c_{\mathrm{Cl}}^{\mathrm{dc}}(\tau )=c_{\mathrm{Cl}}^{\mathrm{dc}}(0)-\frac{iT_{\mathrm{Cl}}^{\mathrm{BM}}}{V^{\mathrm{dc}}F}\tau$
	HA	$c_{\mathrm{HA}}=c_{A}-c_{A^{-}}$

^1^ sodium ion constant flux; ^2^ sodium and hydrogen competitive flux through cation-exchange membrane. The resulting Equation must be solved numerically to find sodium concentration.

## Appendix B

**Table A2:** Composition of the tap water of the Krasnodar.

Parameter	Measured Value
pH	8.0
Specific conductivity, mS/cm	0.704
Sodium concentration, mmol/L	7.43
Chloride concentration, mmol/L	1.26
Sulfate concentration, mmol/L	1.29
Hydrocarbonate concentration, mmol/L	3.59

## Appendix C

**Table A3:** Properties of pilot scale electrodialysis module.

Parameter	Value
Membrane channel dimensions:	
length *L*, cm	40
width *w*, cm	10
intermembrane distance *h*, mm	0,9
Number of elementary cells *n*, pcs	25
Solution flow path	Parallel co-flow
Elementary cell configuration	Two-chamber (BPM-CEM)
Materials:	
Intermembrane separators	HDPE
Separators-turbulazers	HDPE mesh, rectangular cells, cell size 4 × 4 mm, placed at 45° to the flow of solution
cathode	Ti/Pt
anode	Ti/Pt

## References

[1] Finlay K., Bogard M.J. (2022). pH of Inland Waters. Encycl. Inl. Waters.

[2] Berry R.E., Wagner C.J., Haven W. (1973). Method for Preparing Dehydrated Deacidic Citrus Juice Product. U.S. Patent.

[3] Sofralab F. (1984). Deacidification of Food Liquids. U.S. Patent.

[4] Salmon M. (1983). Acidulation of Milk. U.S. Patent.

[5] Bailly M. (2002). Production of organic acids by bipolar electrodialysis: Realizations and perspectives. Desalination.

[6] Bailly M., Balmann H.R., Aimar P., Lutin F., Cheryan M. (2001). Production processes of fermented organic acids targeted around membrane operations: Design of the concentration step by conventional electrodialysis. J. Membr. Sci..

[7] Ibeas V., Correia A.C., Jordão A.M. (2015). Wine tartrate stabilization by different levels of cation exchange resin treatments: Impact on chemical composition, phenolic profile and organoleptic properties of red wines. Food Res. Int..

[8] Kontogiannopoulos K.N., Patsios S.I., Karabelas A.J. (2016). Tartaric acid recovery from winery lees using cation exchange resin: Optimization by Response Surface Methodology. Sep. Purif. Technol..

[9] Gomez Benítez J., Palacios Macías V.M., Sánchez Pazo J.A., Pérez Rodriguez L. (2002). Industrial development of proton exchange for tartrate stabilization of sherry wines. Eur. Food Res. Technol..

[10] Lasanta C., Caro I., Pérez L. (2013). The influence of cation exchange treatment on the final characteristics of red wines. Food Chem..

[11] Vera E., Dornier M., Ruales J., Vaillant F., Reynes M. (2003). Comparison between different ion exchange resins for the deacidification of passion fruit juice. J. Food Eng..

[12] Li N., Wei Y., Li X., Wang J.J., Zhou J., Wang J.J. (2019). Optimization of deacidification for concentrated grape juice. Food Sci. Nutr..

[13] Rialland J.-P., Barbier J.-P. (1984). Procede de Traitement du Lait par une Resine Echangeuse de Cations en Vue de la Fabrication de la Caseine et du Lactoserum. France Patent.

[14] Wang Y., Jiang C., Bazinet L., Xu T. (2019). Electrodialysis-Based Separation Technologies in the Food Industry. Sep. Funct. Mol. Food Membr. Technol..

[15] El Rayess Y., Mietton-Peuchot M. (2016). Membrane Technologies in Wine Industry: An Overview. Crit. Rev. Food Sci. Nutr..

[16] Pismenskaya N., Bdiri M., Sarapulova V., Kozmai A., Fouilloux J., Baklouti L., Larchet C., Renard E., Dammak L. (2021). A Review on Ion-Exchange Membranes Fouling during Electrodialysis Process in Food Industry, Part 2: Influence on Transport Properties and Electrochemical Characteristics, Cleaning and Its Consequences. Membranes.

[17] Liu G., Wu D., Chen G., Halim R., Liu J., Deng H. (2021). Comparative study on tartaric acid production by two-chamber and three-chamber electro-electrodialysis. Sep. Purif. Technol..

[18] Simons R. (1984). Electric field effects on proton transfer between ionizable groups and water in ion exchange membranes. Electrochim. Acta.

[19] Simons R. (1985). Water splitting in ion exchange membranes. Electrochim. Acta.

[20] Zabolotskii V.I., Shel’deshov N.V., Gnusin N.P., Shel N.V., Gnusin N.P., Shel’deshov N.V., Gnusin N.P. (1988). Dissociation of Water Molecules in Systems with Ion-exchange Membranes. Russ. Chem. Rev..

[21] Pärnamäe R., Mareev S., Nikonenko V., Melnikov S., Sheldeshov N., Zabolotskii V., Hamelers H.V.M., Tedesco M. (2021). Bipolar membranes: A review on principles, latest developments, and applications. J. Membr. Sci..

[22] Xue Y., Wang N., Huang C., Cheng Y., Xu T. (2009). Catalytic water dissociation at the intermediate layer of a bipolar membrane: The role of carboxylated Boltorn^®^ H30. J. Membr. Sci..

[23] Giesbrecht P.K., Freund M.S., Giesbrecht P.K., Freund M.S., Giesbrecht P.K., Freund M.S., Giesbrecht P.K., Freund M.S. (2020). Recent Advances in Bipolar Membrane Design and Applications. Chem. Mater..

[24] Bazinet L., Lamarche F., Ippersiel D. (1998). Bipolar-membrane electrodialysis: Applications of electrodialysis in the food industry. Trends Food Sci. Technol..

[25] Tongwen X. (2002). Electrodialysis processes with bipolar membranes (EDBM) in environmental protection—A review. Resour. Conserv. Recycl..

[26] Quoc A.L., Mondor M., Lamarche F., Ippersiel D., Bazinet L., Makhlouf J. (2006). Effect of a combination of electrodialysis with bipolar membranes and mild heat treatment on the browning and opalescence stability of cloudy apple juice. Food Res. Int..

[27] Lin Teng Shee F., Bazinet L. (2009). Cationic balance and current efficiency of a three-compartment bipolar membrane electrodialysis system during the preparation of chitosan oligomers. J. Membr. Sci..

[28] Mier M.P., Iba R., Ortiz I. (2008). Influence of process variables on the production of bovine milk casein by electrodialysis with bipolar membranes. Biochem. Eng. J..

[29] Merkel A., Ashrafi A.M., Ečer J. (2018). Bipolar membrane electrodialysis assisted pH correction of milk whey. J. Membr. Sci..

[30] Serre E., Rozoy E., Pedneault K., Lacour S., Bazinet L. (2016). Deacidification of cranberry juice by electrodialysis: Impact of membrane types and configurations on acid migration and juice physicochemical characteristics. Sep. Purif. Technol..

[31] Rozoy E., Boudesocque L., Bazinet L. (2015). Deacidification of cranberry juice by electrodialysis with bipolar membranes. J. Agric. Food Chem..

[32] Lei C., Li Z., Gao Q., Fu R., Wang W., Li Q., Liu Z. (2020). Comparative study on the production of gluconic acid by electrodialysis and bipolar membrane electrodialysis: Effects of cell configurations. J. Membr. Sci..

[33] Hülber-Beyer É., Bélafi-Bakó K., Nemestóthy N. (2021). Low-waste fermentation-derived organic acid production by bipolar membrane electrodialysis—An overview. Chem. Pap..

[34] Prochaska K., Woźniak-Budych M.J. (2014). Recovery of fumaric acid from fermentation broth using bipolar electrodialysis. J. Membr. Sci..

[35] Tongwen X., Weihua Y., Xu T., Weihua Y., Tongwen X., Weihua Y. (2002). Citric acid production by electrodialysis with bipolar membranes. Chem. Eng. Process. Process Intensif..

[36] Shang Z., Hossain M.M., Wycisk R., Pintauro P.N. (2022). Poly(phenylene sulfonic acid)-expanded polytetrafluoroethylene composite membrane for low relative humidity operation in hydrogen fuel cells. J. Power Sources.

[37] Melnikov S., Bondarev D., Nosova E., Melnikova E., Zabolotskiy V. (2020). Water Splitting and Transport of Ions in Electromembrane System with Bilayer Ion-Exchange Membrane. Membranes.

[38] Kollsman P. (1966). Antipolarization Membrane Having Anionic and Cationic Areas. U.S. Patent.

[39] Leitz F.B. (1971). Cationic-Anionic Ion-Exchange Membrane. U.S. Patent.

[40] Antonov Y.A., Ponomarev M.I., Teselkin V.V., Grebenyk V.D. (1983). Production of alkali with simultaneous water desaltination in electrodialyzer with semibipolar membranes. Chem. Technol. Water.

[41] Shendrik O.R., Ponomarev M.I., Volkov S.A., Grebenyk V.D. (1985). Development and properties of cation-exchange membranes modified with electrosedated dispersion of anionite. Chem. Technol. Water.

[42] Shendrik O.R., Ponomarev M.I., Grebenyk V.D. (1986). Modification of monopolar ion exchange membranes for hydrogen and hydroxyl ions generation. J. Appl. Chem..

[43] Russell B.H., Samuel S.A. (1989). Novel Bipolar Membranes and Process of Manufacture. U.S. Patent.

[44] Strathmann H., Krol J.J., Rapp H.-J.J., Eigenberger G. (1997). Limiting current density and water dissociation in bipolar membranes. J. Membr. Sci..

[45] Balster J.H., Sumbharaju R., Srikantharajah S., Pünt I., Stamatialis D.F., Jordan V., Wessling M. (2007). Asymmetric bipolar membrane: A tool to improve product purity. J. Membr. Sci..

[46] Xu T., Yang W., He B. (2001). A simple model to determine the trends of electric field enhanced water dissociation in a bipolar membrane. Chinese J. Chem. Eng..

[47] Xu T. (2002). Effect of asymmetry in a bipolar membrane on water dissociation—A mathematical analysis. Desalination.

[48] Xu T., Fu R. (2006). A simple model to determine the trends of electric-field-enhanced water dissociation in a bipolar membrane. II. Consideration of water electrotransport and monolayer asymmetry. Desalination.

[49] Zabolotskii V.I., Sheldeshov N.V., Melnikov S.S. (2013). Effect of cation-exchange layer thickness on electrochemical and transport characteristics of bipolar membranes. J. Appl. Electrochem..

[50] Melnikov S.S., Nosova E.N., Melnikova E.D., Zabolotsky V.I. (2021). Reactive separation of inorganic and organic ions in electrodialysis with bilayer membranes. Sep. Purif. Technol..

[51] Gmar S., Chagnes A., Ben Salah Sayadi I., Fauvarque J.F., Tlili M., Ben Amor M. (2016). Semiempirical kinetic modelling of water desalination by electrodialysis processes. Sep. Sci. Technol..

[52] Sadrzadeh M., Kaviani A., Mohammadi T. (2007). Mathematical modeling of desalination by electrodialysis. Desalination.

[53] Zourmand Z., Faridirad F., Kasiri N., Mohammadi T. (2015). Mass transfer modeling of desalination through an electrodialysis cell. Desalination.

[54] Karimi L., Ghassemi A. (2016). An empirical/theoretical model with dimensionless numbers to predict the performance of electrodialysis systems on the basis of operating conditions. Water Res..

[55] Nikonenko V., Nebavsky A., Mareev S., Kovalenko A., Urtenov M., Pourcelly G. (2018). Modelling of Ion Transport in Electromembrane Systems: Impacts of Membrane Bulk and Surface Heterogeneity. Appl. Sci..

[56] Severin B.F., Hayes T.D. (2022). A Michaelis-Menten rate model for the electrodialysis of concentrated salts. Sep. Purif. Technol..

[57] Sadrzadeh M., Mohammadi T., Ivakpour J., Kasiri N. (2008). Separation of lead ions from wastewater using electrodialysis: Comparing mathematical and neural network modeling. Chem. Eng. J..

[58] Sadrzadeh M., Ghadimi A., Mohammadi T. (2009). Coupling a mathematical and a fuzzy logic-based model for prediction of zinc ions separation from wastewater using electrodialysis. Chem. Eng. J..

[59] Wang X., Han X., Zhang X., Li Q., Xu T. (2017). Modeling of Potassium Sulfate Production from Potassium Chloride by Electrodialytic Ion Substitution. ACS Sustain. Chem. Eng..

[60] Kaláb J., Palatý Z. (2012). Electrodialysis of oxalic acid: Batch process modeling. Chem. Pap..

[61] Koter S., Kultys M., Gilewicz-Łukasik B. (2011). Modeling the electric transport of HCl and H_3_PO_4_ mixture through anion-exchange membranes. Membr. Water Treat..

[62] Koter S. (2008). Separation of weak and strong acids by electro-electrodialysis—Experiment and theory. Sep. Purif. Technol..

[63] Pismenskiy A., Nikonenko V., Urtenov M., Pourcelly G. (2006). Mathematical modelling of gravitational convection in electrodialysis processes. Desalination.

[64] Urtenov M.K., Uzdenova A.M., Kovalenko A.V., Nikonenko V.V., Pismenskaya N.D., Vasil’eva V.I., Sistat P., Pourcelly G. (2013). Basic mathematical model of overlimiting transfer enhanced by electroconvection in flow-through electrodialysis membrane cells. J. Membr. Sci..

[65] Nikonenko V., Urtenov M., Mareev S., Pourcelly G. (2020). Mathematical Modeling of the Effect of Water Splitting on Ion Transfer in the Depleted Diffusion Layer Near an Ion-Exchange Membrane. Membranes.

[66] Mareev S.A., Evdochenko E., Wessling M., Kozaderova O.A., Niftaliev S.I., Pismenskaya N.D., Nikonenko V.V. (2020). A comprehensive mathematical model of water splitting in bipolar membranes: Impact of the spatial distribution of fixed charges and catalyst at bipolar junction. J. Membr. Sci..

[67] Ortega A., Arenas L.F., Pijpers J.J.H., Vicencio D.L., Martínez J.C., Rodríguez F.A., Rivero E.P. (2022). Modelling water dissociation, acid-base neutralization and ion transport in bipolar membranes for acid-base flow batteries. J. Membr. Sci..

[68] Wang Y., Wang A., Zhang X., Xu T. (2011). Simulation of Electrodialysis with Bipolar Membranes: Estimation of Process Performance and Energy Consumption. Ind. Eng. Chem. Res..

[69] Volgin V.M., Davydov A.D. (2005). Ionic transport through ion-exchange and bipolar membranes. J. Membr. Sci..

[70] Mier M.P., Ibañez R., Ortiz I. (2008). Influence of ion concentration on the kinetics of electrodialysis with bipolar membranes. Sep. Purif. Technol..

[71] Bui J.C., Digdaya I., Xiang C., Bell A.T., Weber A.Z. (2020). Understanding Multi-Ion Transport Mechanisms in Bipolar Membranes. ACS Appl. Mater. Interfaces.

[72] Chen Y., Baygents J.C., Gervasio D., Farrell J. (2021). Factors Affecting Hydroxide Ion Concentrations in Bipolar Membranes. J. Membr. Sci. Res..

[73] Mazrou S., Kerdjoudj H., Chérif A.T., Elmidaoui A., Molénat J. (1998). Regeneration of hydrochloric acid and sodium hydroxide with bipolar membrane electrodialysis from pure sodium chloride. New J. Chem..

[74] Balmann H.R., Bailly M., Lutin F., Aimar P., Roux-de Balmann H., Bailly M., Lutin F., Aimar P. (2002). Modelling of the conversion of weak organic acids by bipolar membrane electrodialysis. Desalination.

[75] Koter S. (2007). Modeling of weak acid production by the EDB method. Sep. Purif. Technol..

[76] Jaime-Ferrer J.S., Couallier E., Viers P., Rakib M. (2009). Two-compartment bipolar membrane electrodialysis for splitting of sodium formate into formic acid and sodium hydroxide: Modelling. J. Membr. Sci..

[77] Zabolotskii V.I., Utin S.V., Shel’deshov N.V., Lebedev K.A., Vasilenko P.A. (2011). Correction of pH of diluted solutions of electrolytes by electrodialysis with bipolar membranes. Russ. J. Electrochem..

[78] Zabolotskii V.I., Utin S.V., Lebedev K.A., Vasilenko P.A., Shel’Deshov N.V. (2012). Study of pH correction process of chloride-bicarbonate dilute solutions by electrodialysis with bipolar membranes. Russ. J. Electrochem..

[79] Sheldeshov N.V., Zabolotsky V.I., Kovalev N.V., Karpenko T.V. (2020). Electrochemical characteristics of heterogeneous bipolar membranes and electromembrane process of recovery of nitric acid and sodium hydroxide from sodium nitrate solution. Sep. Purif. Technol..

[80] Polyanskiy N.G., Gorbunov G.V., Polyanskaya N.L. (1976). Methods for Ion-exchangers Properties Investigation. Chemistry.

[81] Zabolotsky V.I., Achoh A.R., Lebedev K.A., Melnikov S.S. (2020). Permselectivity of bilayered ion-exchange membranes in ternary electrolyte. J. Membr. Sci..

[82] Corless R.M., Gonnet G.H., Hare D.E.G., Jeffrey D.J., Knuth D.E. (1996). On the Lambert W function. Adv. Comput. Math..

[83] Zabolotskii V.I., Manzanares J.A., Mafe S., Nikonenko V.V., Lebedev K.A. (2002). Steady-state Ion Transport through a Three-Layered Membrane System: A Mathematical Model Allowing for Violation of the Electroneutrality Condition. Russ. J. Electrochem..

[84] Vasilenko P.A., Utin S.V., Zabolotskiy V.I., Lebedev K.A. (2017). Mathematical model of the correction of ph softened water in a long channel of electrodialysis with bipolar membrane. Polythemat. Online Sci. J. Kuban State Agrar. Univ..

[85] Nikonenko V.V., Pismenskaya N.D., Istoshin A.G., Zabolotsky V.I., Shudrenko A.A. (2008). Description of mass transfer characteristics of ED and EDI apparatuses by using the similarity theory and compartmentation method. Chem. Eng. Process. Process Intensif..

[86] Zabolotskii V.I., Melnikov S.S., Demina O.A. (2014). Prediction of the mass exchange characteristics of industrial electrodialyzer concentrators. Russ. J. Electrochem..

[87] Tanaka Y. (2003). Mass transport and energy consumption in ion-exchange membrane electrodialysis of seawater. J. Membr. Sci..

[88] Tanaka Y., Ehara R., Itoi S., Goto T. (2003). Ion-exchange membrane electrodialytic salt production using brine discharged from a reverse osmosis seawater desalination plant. J. Membr. Sci..

[89] Tanaka Y. (2011). Ion-exchange membrane electrodialysis for saline water desalination and its application to seawater concentration. Ind. Eng. Chem. Res..

[90] Melnikov S., Loza S., Sharafan M., Zabolotskiy V. (2016). Electrodialysis treatment of secondary steam condensate obtained during production of ammonium nitrate. Technical and economic analysis. Sep. Purif. Technol..

[91] Melnikov S.S., Sheldeshov N.V., Zabolotsky V.I., Loza S., Achoh A. (2017). Pilot scale complex electrodialysis technology for processing a solution of lithium chloride containing organic solvents. Sep. Purif. Technol..

